# The complete chloroplast genome of the lipstick tree, *Bixa Orellana* (Bixaceae)

**DOI:** 10.1080/23802359.2018.1535847

**Published:** 2018-11-21

**Authors:** Seping Dai, Xuwen Li, Jianzhong Ni, Lin Ruan, Renchao Zhou, Wei Lun Ng

**Affiliations:** a Guangzhou Institute of Forestry and Landscape Architecture, Guangzhou, China;; b State Key Laboratory of Biocontrol and Guangdong Provincial Key Laboratory of Plant Resources School of Life Science, Sun Yat-sen University, Guangzhou, China

**Keywords:** *Bixa*, achiote, natural dye, complete chloroplast genome, automated assembly

## Abstract

*Bixa orellana* is a small tree known for its red, oil-soluble pigment contained in the seed coat that is used as a natural dye and food coloring. In this study, we assembled and characterized the complete chloroplast genome of *B. orellana* as a resource for future genetic studies. With a total length of 159,825 bp, the chloroplast genome comprised of a large single-copy (LSC) region of 89,476 bp, a small single-copy (SSC) region of 19,617 bp, and two inverted repeat (IR) regions of 25,356 bp each. A total of 127 genes were predicted, consisting of 83 protein-coding genes, 36 tRNA genes, and 8 rRNA genes. Phylogenetic analysis confirmed the position of *B. orellana* within the order Malvales.

*Bixa orellana* Linnaeus is an evergreen shrub or small tree known for its red, oil-soluble pigment (bixin) that is contained in the seed coat. As one of the five-member species of the genus, it is native to tropical America but widely cultivated in tropical regions of the world (eFloras 2008). Aside from the red pigment from the seed that is usually used as natural dyes and food coloring, the different parts of the plant are also used as traditional medicine in many cultures (de Araujo Vilar et al. 2014). As the best-known species in the family Bixaceae, in this study, we characterized the complete chloroplast genome sequence of *B. orellana* as a resource for future genetic studies on this and other related species.

Leaf samples of *B. orellana* were obtained from the Guangzhou Institute of Forestry and Landscape Architecture, with voucher specimen also deposited at the Sun Yat-sen University Herbarium (SYS; specimen code SYS-Bore-2018-01). After DNA extraction, a library with the insertion size of ∼400 bp was constructed, and high-throughput DNA sequencing (pair-end 150 bp) was performed on an Illumina Hiseq 2500 platform, generating approximately 4 Gb of sequence data. Using a *B. orellana* partial *rbc*L gene sequence (Y15139.1) as seed and the *Gossypium arboretum* chloroplast genome sequence (HQ325740.1) as reference, the chloroplast genome was assembled from the Illumina data using the program NOVOPlasty (Dierckxsens et al. 2017). The assembled chloroplast genome sequence was then annotated using DOGMA (Wyman et al. 2004) and manually corrected.

The complete chloroplast genome sequence of *B. orellana* (GenBank accession MH751592) was 159,825 bp in length, with a large single-copy (LSC) region of 89,476 bp, a small single-copy (SSC) region of 19,617 bp, and two inverted repeat (IR) regions of 25,356 bp each. A total of 127 genes were predicted, consisting of 83 protein-coding genes, 36 tRNA genes, and 8 rRNA genes. The overall GC content was 36.40%.

To understand the phylogenetic position of *Bixa* within the order Malvales, chloroplast genome sequences of eight other representative species in Malvales (*Aquilaria yunnanensis*, *Bombax ceiba*, *Daphne kiusiana*, *Firmiana major*, *Gossypium arboretum*, *Heritiera angustata*, *Theobroma cacao*, *Tilia oliveri*), as well as *Melastoma candidum* of the order Myrtales, were downloaded from the NCBI GenBank database for phylogenetic analysis. The sequences were aligned using MAFFT v7.307 (Katoh and Standley 2013), and RAxML (Stamatakis 2014) was used to construct a maximum likelihood tree with *M. candidum* as outgroup. As shown in Figure 1, *B. orellana* clustered within Malvales with strong bootstrap support.

**Figure 1.**
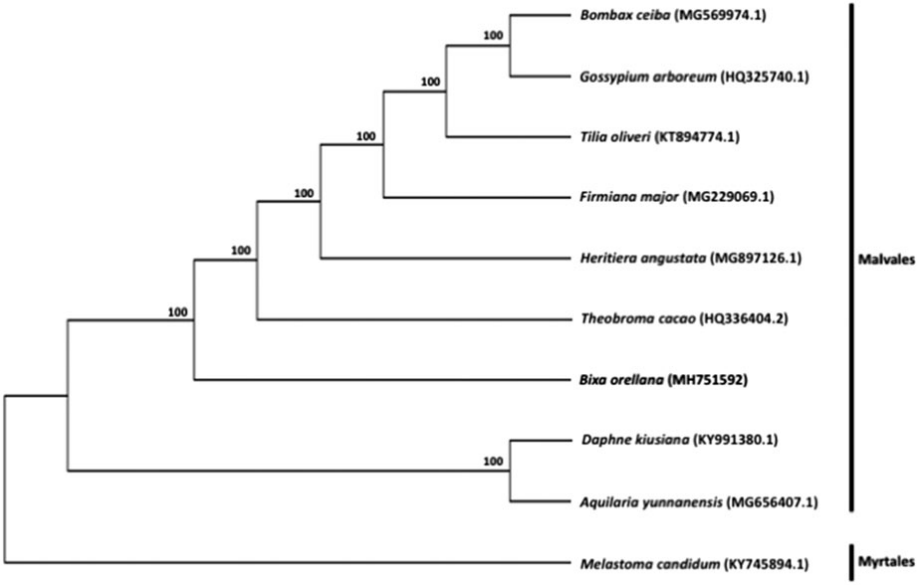
Maximum likelihood tree showing the relationship among *Bixa orellana* and representative species within the order Malvales, based on whole chloroplast genome sequences, with *Melastoma candidum* (Myrtales) as outgroup. Shown next to the nodes are bootstrap support values based on 1000 replicates.

## Disclosure statement

No potential conflict of interest was reported by the authors.
